# The relationship between symptom burden and systemic inflammation differs between male and female athletes following concussion

**DOI:** 10.1186/s12865-020-0339-3

**Published:** 2020-03-12

**Authors:** Alex P. Di Battista, Nathan Churchill, Shawn G. Rhind, Doug Richards, Michael G. Hutchison

**Affiliations:** 1 Faculty of Kinesiology & Physical Education, University of Toronto, 55 Harbord St., Toronto, ON M5S 2W6 Canada; 2 Defence Research and Development Canada, Toronto Research Centre, Toronto, ON Canada; 3 Neuroscience Program, Keenan Research Centre for Biomedical Science of St. Michael’s Hospital, Toronto, ON Canada; 4 David L. MacIntosh Sport Medicine Clinic, Faculty of Kinesiology & Physical Education, University of Toronto, Toronto, ON Canada; 5 Keenan Research Centre for Biomedical Science of St. Michael’s Hospital, Toronto, ON Canada

**Keywords:** Biomarkers, Inflammation, mTBI, Multivariate statistics, Immunity

## Abstract

**Background:**

Inflammation appears to be an important component of concussion pathophysiology. However, its relationship to symptom burden is unclear. Therefore, the purpose of this study was to evaluate the relationship between symptoms and inflammatory biomarkers measured in the blood of male and female athletes following a sport-related concussion (SRC).

**Results:**

Forty athletes (*n* = 20 male, n = 20 female) from nine interuniversity sport teams at a single institution provided blood samples within one week of an SRC. Twenty inflammatory biomarkers were quantitated by immunoassay. The Sport Concussion Assessment Tool version 5 (SCAT-5) was used to evaluate symptoms. Partial least squares (PLS) analyses were used to evaluate the relationship(s) between biomarkers and symptoms. In males, a positive correlation between interferon (IFN)-γ and symptom severity was observed following SRC. The relationship between IFN-γ and symptoms was significant among all symptom clusters, with cognitive symptoms displaying the largest effect. In females, a significant negative relationship was observed between symptom severity and cytokines IFN-γ, tumor necrosis factor (TNF)-α, and myeloperoxidase (MPO); a positive relationship was observed between symptom severity and MCP-4. Inflammatory mediators were significantly associated with all symptom clusters in females; the somatic symptom cluster displayed the largest effect.

**Conclusion:**

These results provide supportive evidence of a divergent relationship between inflammation and symptom burden in male and female athletes following SRC. Future investigations should be cognizant of the potentially sex-specific pathophysiology underlying symptom presentation.

## Background

Sport-related concussion (SRC) is a complex injury that can lead to somatic, cognitive, visual, sleep and emotional disturbances. Symptoms typically abate within weeks but can persist for months to years in a subset of individuals [1]. Importantly, symptom burden is a key tool for clinicians in guiding patients through the recovery process; the resolution of symptoms is a prerequisite for medical clearance and return to sport participation [1]. While the biological mechanisms underlying symptom presentation following injury remain elusive, the gap in our understanding comes as no surprise, as there is significant heterogeneity in symptom presentation between individuals, both in severity and type. In addition, symptoms commonly observed following SRC have also been reported in a variety of other conditions such as polytrauma, infection, and mental illness [2–5]. In views of this, inflammation presents as a unifying concept, as it has not only been associated with concussion-like symptoms across numerous medical conditions [5], but is increasingly recognized as an important and prominent feature of concussion secondary injury [6–10]. Therefore, investigating the relationship between inflammation and symptom burden following concussion may help elucidate clinically meaningful pathophysiological mechanisms that mediate patient recovery [11, 12].

Rathbone and colleagues suggested inflammation as a common mechanism underlying the constellation of symptoms observed following concussion [5]. This assertion was made in light of the evidence linking inflammatory mediators to numerous symptoms commonly observed following concussion that are seen in medical maladies such as headache, chronic fatigue syndrome, in response to immunomodulatory medical treatments, and psychological conditions such as depression and anxiety [5]. Importantly, evidence of these relationships was commonly characterized by the presence of symptom(s) and altered blood concentrations (typically an increase) of inflammatory cytokines and/or chemokines [5].

Systemic inflammatory mediators can communicate with the central nervous system (CNS) via several mechanisms. Primary afferent and efferent nerves innervating the CNS can respond to cytokine and chemokine signalling from either the brain or periphery [13, 14], CNS-derived neuroendocrine hormones can interact with their respective receptors on circulating leukocytes in the periphery [15–18], and the blood brain barrier (BBB) permits passage of leukocytes and their mediators (cytokines and chemokines) via receptor-mediated transport, endothelial transmigration, and/or diffusion [19]. As a result of this bi-directional communicative network, peripheral inflammatory indices may be used to 1) evaluate the systemic consequences of brain-related maladies, or 2) indirectly elucidate processes occurring in the CNS. An example of this can be seen in sickness behaviour, whereby infection or illness results in fatigue, malaise, decreased appetite, lack of concentration, and feelings of depression and lethargy [20–22]. This constellation of behavioural and physiological changes is due primarily to systemic and centrally produced cytokines, namely tumor necrosis factor (TNF)-α, interleukins (IL)-1β, IL-6, and interferons (IFNs) [13, 22–26].

Our group recently identified a unique inflammatory profile in the peripheral blood of athletes following SRC that was distinct from non-head injury and characterized by elevations in the circulating chemokines monocyte chemoattractant protein (MCP)-4 and macrophage inflammatory protein (MIP)-1β [6]. In addition, higher concentrations of MCP-1 and -4 were associated with prolonged recovery [6]. Similarly, altered inflammatory gene expression in leukocytes [8, 9], and increased brain extracellular vesicles containing TNF-α and IL-8 have also been observed in the peripheral blood of subjects following SRC [27]. Despite these findings and the strong linkage between inflammation and symptomology across numerous clinical conditions, human data characterizing the potential role of inflammation on symptom burden following concussion is scarce. Su and colleagues observed that a higher concentration of the circulating inflammatory index C-reactive protein (CRP) was associated with a greater symptom burden in subjects in the months following mTBI [28]. Likewise, Nitta and colleagues recently identified a positive relationship between serum interleukin IL-6 concentrations and symptom duration following SRC [10]. While this is intriguing, the relationship between concussion symptoms and inflammation has yet to be evaluated across the range of inflammatory mediators previously studied in TBI [6, 29–31]. Furthermore, probable sex differences have not been accounted for; despite the profound differences observed in male versus female immune responses as a consequence of genetic, environment and hormonal influences [32, 33], few studies evaluating inflammation stratify their cohorts by sex [33].

The present study builds upon prior works by examining the relationships between symptom burden and inflammation in males and females following SRC. We hypothesized that symptoms reported in the subacute period following injury would correlate with systemic inflammatory cytokines and chemokines, and that this relationship would differ between males and females. This hypothesis was evaluated in a cohort of university-level athletes assessed within seven days post-injury using a set of multivariate PLS models. These analyses were performed with the goal of improving our understanding of how inflammation may mediate symptom burden in males and females following SRC.

## Results

### Athlete characteristics and biomarker values

**Fig. 1:**
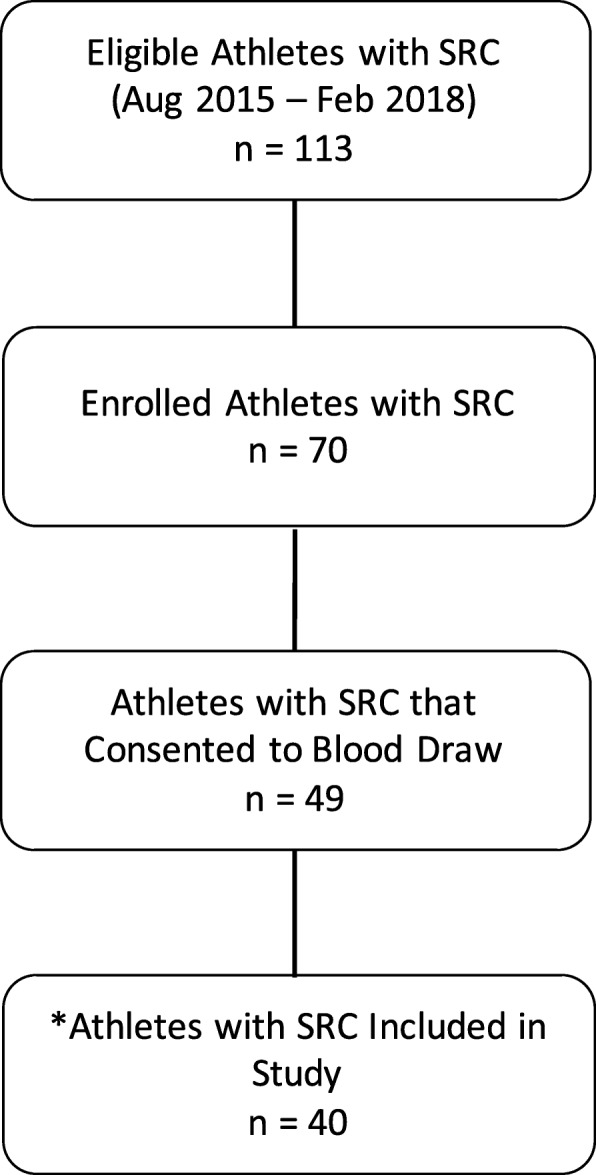
Athlete enrollment from the objective measures of sport concussion project, and study participant selection. SRC = sport-related concussion. * Nine athletes who consented to provide blood were lost for various reasons: *n* = 3 were lost due to no SCAT5 data, *n* = 2 were lost due to the use of prescribed medications that potentially interfere with the inflammatory response, and *n* = 4 were lost due to an inability to acquire a blood sample

Athlete demographics, symptoms, and recovery time can be found in Table 1, and participant recruitment and enrollment information can be found in Fig. 1. There were no significant differences in any of the clinical characteristics between male and female athletes; notably, total symptom reporting (*p* = 0.522), symptom severity (*p* = 0.763), and the median days to medical clearance (*p* = 0.513) were similar. Male and female biomarker values following SRC can be observed in Table 2. While there were no significant differences in biomarker concentrations between males and females after correcting for multiple comparisons, uncorrected MCP-1 (66.6 vs. 53.3 pg/mL, *p* = 0.023), MIP-1B (43.6 vs 31.8 pg/mL, *p* = 0.007) and Eotaxin (94.2 vs. 81 pg/mL, *p* = 0.028) concentrations were significantly lower in females compared to males, and IP-10 (179.4 vs 207.7 pg/mL, *p* = 0.026) concentrations were significantly higher in females compared to males.

**Table 1:** Characteristics of Athletes with SRC

Variable	Males (n = 20)	Females (***n*** = 20)
Age	21.4 (19.9–22.3)	20.4 (18.9–22)
Concussion History (n, %)	12 (60)	11 (55)
Sport (*n*, %)		
Basketball	1 (5)	1 (5)
Field hockey	–	2 (10)
Football	6 (30)	–
Ice hockey	5 (25)	5 (25)
Lacrosse	1 (5)	3 (15)
Mountain biking	–	1 (5)
Rugby	5 (25)	6 (30)
Soccer	–	1 (5)
Volleyball	2 (10)	1 (5)
Days from injury to assessment	4 (3–5.2)	5 (3–5)
SCAT5 symptoms		
Total Symptoms	9.5 (4.8–17.2)	9.0 (5.8–12.2)
Symptom Severity	16.0 (4.8–43.5)	14.5 (6.8–23.5)
Symptom Clusters		
Somatic	9.5 (2.8–17.5)	10 (3.8–14)
Cognitive	6 (2.5–14)	3.0 (1.8–6.2)
Sleep	1.5 (0–6.5)	2.0 (1–4.2)
Emotion	1 (0–4.0)	0 (0–2.0)
Days to medical clearance	27 (21–46.2)	23 (12–60)

sport-related concussion (SRC); sport concussion assessment tool 5 (SCAT5).

All values reported as the median and interquartile range, unless otherwise stated

**Table 2:** Inflammatory biomarker concentrations in athletes with SRC

Marker	Males (***n*** = 20)	Females (***n*** = 20)	***p*** value	FDR
IFN-γ	3.1 (2.5–5.0)	4.1 (2.5–5.9)	0.326	no
TNF-α	1.7 (1.4–2.2)	1.5 (1.3–1.9)	0.305	no
MPO (ng/mL)	13.4 (7.5–21.4)	9.0 (7.9–11.4)	**0.018**	no
IL-8	2.0 (1.7–2.6)	2.0 (1.3–2.4)	0.149	no
MCP-1	66.6 (57.6–73.5)	53.3 (45.8–65.4)	0.090	no
MCP-4	27.5 (20.5–32.4)	23.0 (18.9–29.1)	0.505	no
MIP-1β	43.6 (31.5–53.6)	31.8 (25.0–40.0)	**0.021**	no
IP-10	179.4 (133.2–219.8)	207.7 (166.4–294.8)	**0.016**	no
TARC	72.7 (44.3–94.9)	55.1 (48.2–67.5)	0.061	no
Eotaxin	94.2 (82.2–127.5)	81.0 (66.0–94.8)	**0.028**	no

sport-related concussion (SRC); false discovery rate (FDR); interferon (IFN)-γ, tumor necrosis factor (TNF)-α, myeloperoxidase (MPO), interleukin (IL)- 8, monocyte chemoattractant protein (MCP)-1, −4, macrophage inflammatory protein (MIP)-1β, interferon gamma-induced protein (IP)-10, and thymus and activation-regulated chemokine (TARC)

All values reported as the median and interquartile range in pg/mL, unless otherwise stated

*P* values are derived from bootstrap ratios (BSR) of the mean difference between biomarker values in male and female athletes, corrected at FDR = 0.05

In the case of deviations from normality, the BSR was calculated from the winsorized mean difference

Bold values indicate pre-corrected *p* < 0.05

### Relationship between symptoms and days to recovery

A significant, large correlation between calculated symptom severity and days to recovery (i.e., medical clearance) was observed for both males (mean rho = 0.73, *p* < 0.001, BSR = 6.8) and females (mean rho = 0.63, p < 0.001, BSR = 3.5).

### Relationship between inflammatory biomarkers and symptoms

**Fig. 2:**
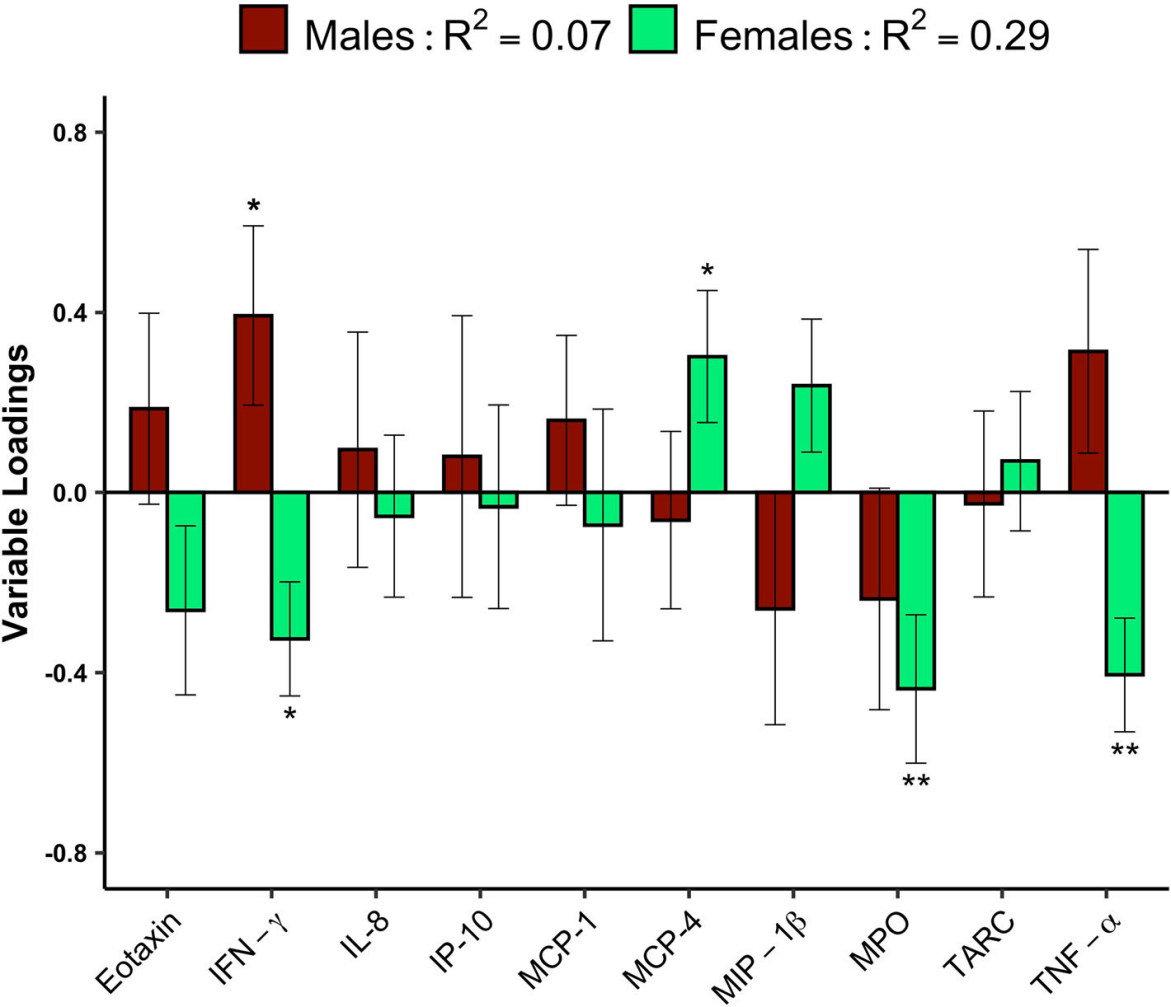
Correlation between Inflammatory biomarkers and symptom severity in male and female athletes following a sport-related concussion. Eotaxin, interferon (IFN)-γ, interleukin (IL)-8, interferon gamma-induced protein (IP)-10, monocyte chemoattractant protein (MCP)-1, − 4, macrophage inflammatory protein (MIP)-1β, Myeloperoxidase (MPO), thymus and activation-regulated chemokine (TARC), and tumor necrosis factor (TNF)-α. Plots show the contributions of biomarkers measured in the subacute period following injury towards symptom severity in male (*n* = 20) and female (n = 20) athletes. Bars represent variable loadings and the standard error derived from bootstrapped resampling (5000 iterations, male = red, female = green). Significance is displayed at *p* < 0.05 *, *p* < 0.01** and *p* < 0.001***

PLS plots showing the relationship between symptom severity and inflammatory biomarkers following SRC can be seen in Fig. 2. The plot depicts biomarker loadings which describe their weighted contribution towards symptom severity, with effect sizes determined via bootstrap ratios (BSR) (see **methods** section). In male athletes, symptom severity significantly positively correlated with blood concentrations of IFN-γ (*p* = 0.04, BSR = 2). Conversely, in female athletes, symptom severity negatively correlated with IFN-γ (*p* = 0.01, BSR = 2.6), MPO (*p* = 0.008, BSR = 2.6), and TNF-α (*p* = 0.001, BSR = 3.2), and positively correlated with MCP-4 (p = 0.04, BSR = 2.1) (Fig. 2). Cross-correlation (R^2^) of the PLS model containing symptom severity and inflammatory biomarkers was 0.07 (95% CI = 0.04–0.1) in males and 0.29 (95% CI = 0.24–0.35) in females.

**Fig. 3:**
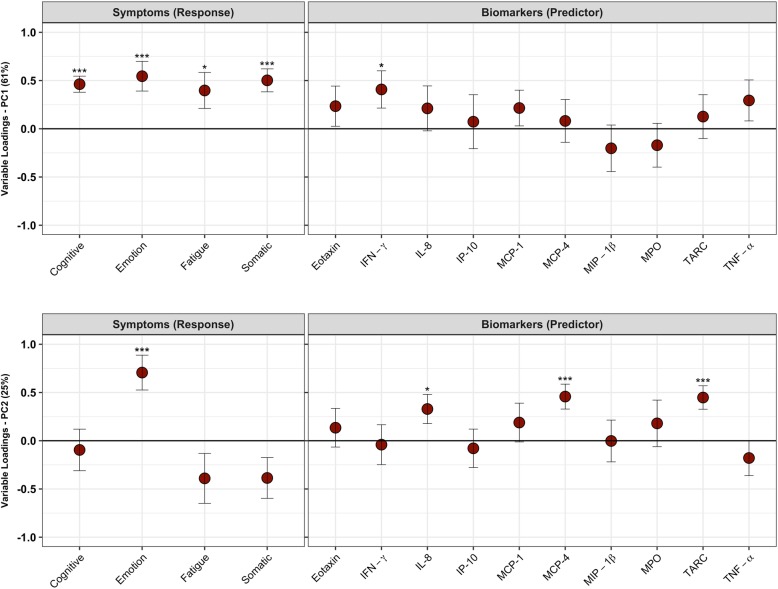
Correlation between Inflammatory biomarkers and symptom clusters in male athletes following sport-related concussion. Tumor necrosis factor (TNF)-α, interferon (IFN)-γ, Myeloperoxidase (MPO), interleukin (IL)-8, monocyte chemoattractant protein (MCP)-1, − 4, macrophage inflammatory protein (MIP)-1β, interferon gamma-induced protein (IP)-10, thymus and activation-regulated chemokine (TARC), and eotaxin. Plots show the correlation between biomarkers measured in the subacute period following injury and symptom clusters on **a)** the first latent variable, and **b)** the second latent variable. Circles represent the variable loadings and standard error derived from bootstrapped resampling. Significance is displayed at *p* < 0.05 *, *p* < 0.01** and *p* < 0.001

**Fig. 4:**
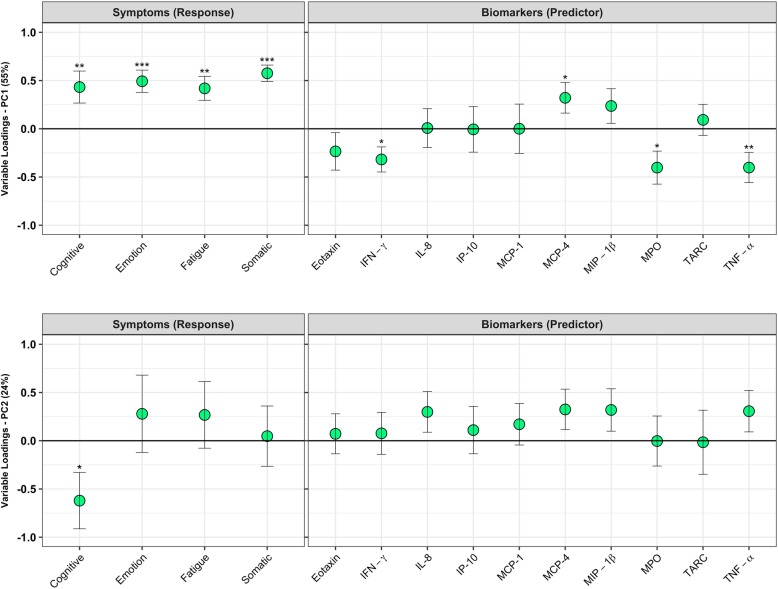
Correlation between Inflammatory biomarkers and symptom clusters in female athletes following sport-related concussion. Tumor necrosis factor (TNF)-α, interferon (IFN)-γ, Myeloperoxidase (MPO), interleukin (IL)-8, monocyte chemoattractant protein (MCP)-1, − 4, macrophage inflammatory protein (MIP)-1β, interferon gamma-induced protein (IP)-10, thymus and activation-regulated chemokine (TARC), and eotaxin. Plots show the correlation between biomarkers measured in the subacute period following injury and symptom clusters on **a**) the first latent variable, and **b**) the second latent variable. Circles represent the variable loadings and standard error derived from bootstrapped resampling. Significance is displayed at *p* < 0.05 *, *p* < 0.01** and *p* < 0.001

PLS plots showing the correlation between symptom clusters and inflammatory biomarkers in male and female athletes following SRC can be found in Figs. 3 & 4. Latent variables are plotted, describing the two most relevant structures describing the relationship between symptoms and biomarkers. In male athletes, there were two latent variables describing 86% of the covariance between symptoms and biomarkers. The first latent variable (LV) (explaining 61% of covariance between symptoms and biomarkers) was characterized by a significant positive correlation between all symptom clusters and IFN-γ (*p* = 0.03, BSR = 2.1); the cognitive symptom cluster displayed the greatest effect size (*p* < 0.001, BSR = 5.5), while the fatigue symptom cluster displayed the lowest (*p* = 0.03, BSR = 2.1) (Fig. 3a). The second LV, (explaining 25% of covariance) was characterized by a positive correlation between symptoms of emotion (*p* < 0.001, BSR = 3.9) and IL-8 (*p* = 0.03, BSR = 2.2), MCP-4 (*p* < 0.001, BSR = 3.5), and thymus and activation regulated chemokine (TARC) (p < 0.001, BSR = 3.7) (Fig. 3b).

In female athletes, there were two latent variables describing 79% of the covariance between symptoms and biomarkers. The first LV (explaining 55% of covariance) was characterized by a negative relationship between symptom clusters and inflammatory biomarkers: Higher symptoms (all clusters) were negatively correlated with TNF-α (*p* = 0.01, BSR = 2.6), IFN-γ (*p* = 0.01, BSR = 2.4) and myeloperoxidase (MPO) (*p* = 0.02, BSR = 2.3). However, a positive correlation was observed between symptoms (all clusters) and MCP-4 (*p* = 0.04, BSR = 2.0). The largest effects were seen with somatic symptoms (*p* < 0.001, BSR = 6.8), while the smallest effects were seen with cognitive symptoms (*p* = 0.009, BSR = 2.6) (Fig. 4a). In the second LV (explaining 24% of covariance), while there was a relationship between cognitive symptoms (*p* = 0.03, BSR = 2.1) and biomarkers, there were no significant associations with individual inflammatory biomarker loadings (Fig. 4b).

## Discussion

In this study, we identified a significant relationship between symptom burden and systemic inflammation following SRC, with differences between males and females. Within the first week of injury, reported symptom severity was inversely correlated with inflammatory cytokines in the peripheral blood of female athletes, yet positively correlated in males. Importantly, these differences were noted despite male and female groups having a comparable symptom burden and time to recovery. This suggests that inflammation is an important and clinically relevant component of secondary injury following SRC but may present differently in males and females.

We observed greater symptom burden was associated with lower concentrations of the classical inflammatory cytokines IFN-γ and TNF-α, and the innate immune function marker MPO in female athletes following SRC; a positive relationship was observed with the chemokine MCP-4. The somatic symptom cluster displayed the greatest effect, while the cognitive cluster displayed the lowest, and the overall relationship between symptoms and inflammation was comparatively more generalizable in females than males (PLS cross-correlation R^2^ = 0.07 in males, R^2^ = 0.29 in females). While we hypothesized a sex-difference in the relationship between inflammation and symptom burden following injury, the direction of the association observed in female athletes is seemingly non-intuitive. The preponderance of evidence linking concussion symptoms to inflammation suggests a positive relationship (for review see [5]), and females typically have a greater inflammatory response to challenge compared to males [33]. However, in a recent meta-analysis evaluating peripheral chemokine and cytokines in depression, while the majority of studies found positive relationships, lower concentrations of TNF-α and IFN-γ were also observed (for review see [34]). Furthermore, a number of perturbations to inflammatory genes in peripheral leukocytes have been observed following SRC, displaying both upregulation and downregulation [8, 9]. Hence, it is possible that both the complexity of the inflammatory response to concussion as well as potentially divergent responses in males and females contribute to these mixed findings. In addition, it is conceivable that the menstrual cycle may have had an effect; immune function can vary widely with hormonal changes during the menstrual cycle, with potential immunosuppression in the luteal phase [35–37]. While it is still unclear why we observed that symptom burden was correlated with lower levels of inflammatory cytokines in females following SRC, the results of this investigation provide supportive evidence that inflammation following concussion should not be looked at without dichotomizing groups by sex.

As opposed to females, in the current study symptom severity was positively correlated with blood concentrations of IFN-γ in male athletes following SRC. This observation is generally supported by literature that has shown a positive association between the family of IFN proteins and symptoms such as headache, fatigue, irritability, hostility, and depression [34, 38, 39] in numerous maladies. While we found IFN-γ was associated with all symptom clusters, cognitive symptoms displayed the highest effect; in females cognitive symptoms displayed the lowest effect. Furthermore, we observed a small, stable effect in males between the emotion symptom cluster and the chemokines IL-8, MCP-4 and TARC. These mediators are primarily responsible for neutrophil [40], monocyte [40] and lymphocyte chemoattraction [41], respectively, and chemokines have been broadly implicated in leukocyte trafficking to the brain following TBI [29, 40, 42, 43]; indeed, higher concentrations of IL-8 in the blood acutely following severe TBI have been correlated with unfavorable outcome [30]. Regarding symptom burden, although chemokines have been broadly implicated in depression, the specific relationship(s) between MCP-4, IL-8 and TARC and the behaviours and mood states comprising the “emotion” symptom cluster in the current study (emotional, irritability, sadness, nervous/anxious) are not well defined. Furthermore, it is important not to overstate this relationship, as these findings were found on the second LV comprising only 25% of the covariance in the PLS model and are comparatively small relative to the magnitude of the relationship observed between symptoms and IFN-γ. Taken together, further research is warranted both to replicate these findings and to investigate the mechanisms mediating the relationship between inflammation and symptom burden in male athletes following SRC.

The mechanism(s) underlying the relationship observed in the current study between symptom burden and inflammation following SRC are unknown. However, given the strong influence of the neuroendocrine system on inflammation [44–46], its role in both brain injury [9, 17, 47–49] and related symptomology [50–53] along with the differences in neuroendocrine biology between males and females [54–56], it is plausible that the stress-immune axis mediates the relationship between symptoms and inflammation following concussion. The two major arms of the body’s stress system, the sympathetic nervous system (SNS) and the hypothalamic pituitary adrenal (HPA) axis can augment the immune system in numerous ways. For example, the HPA-axis, through the actions of glucocorticoids can have strong immunosuppressive effects [57–59], while the SNS can be pro- or anti-inflammatory depending on the nature of the stimulus and subsequent catecholamine-adrenergic receptor pairing [15, 32, 31], and have recently observed that neuroendocrine hormones in the blood following sport concussion are associated with both symptom burden and time to medical clearance [60]. In support of the latter, Merchant-Borna and colleagues observed a change in lymphocyte transcription of genes regulating HPA-axis activity and inflammation at seven days post-SRC [9]. However, the complex interactions between sex, neuroendocrine-immune signalling, their effect on symptom burden, and the temporal shifts in these processes that likely occur to restore homeostasis throughout recovery are not understood. Lastly, given the pleiotropicity, biological redundancy, and dynamic nature of the inflammatory response, it is difficult to control for the vast number of confounds when conducting a human study. As a result, a normative healthy group can be a potential source of biological noise in statistical analyses. The purposeful exclusion of a healthy control sample in the current study allowed for a direct evaluation of the relationship between symptom burden and inflammation post-injury. However, this approach also allows for the possibility that pre-injury biological conditions and/or phenotypes affecting the inflammatory system or that contribute to symptom burden, impacted our findings. In view of this, in the current study we observed a relationship between inflammatory biomarkers and concussion history in male athletes following SRC (Supplementary Fig. 1), and it is unclear how this may have impacted post-injury inflammation. Hence future studies aimed at uncovering the intricacies of these interrelationships, including both pre- and post-injury biology and the potential mediating effect of concussion history, are necessary.

The results of the study must be interpreted within the context of its limitations. While the sport concussion assessment tool (SCAT) symptom profile is a well-studied and useful tool utilized by clinicians and concussion researchers, investigation into specific symptoms may have benefited from more directed measures. For example, clinical screening tools for depression (i.e., the Beck Depression Inventory) and anxiety disorders (i.e., the general anxiety disorder questionnaire-7) may have provided utility to the current analysis, as would physiological evaluations of sleep (i.e., heart rate variability monitoring), and more objective neuropsychological evaluations of cognitive function. Furthermore, a larger study sample would have permitted a greater degree of subgroup analysis (i.e., prior concussion history, menstrual cycle in females), and baseline evaluations would have allowed for the consideration of potential pre-injury differences in symptom presentation between males and females [61, 62]. Finally, a more narrow and acute sample timepoint may have helped capture a more profound inflammatory response to injury; despite there being no relationship between the time elapsed from SRC to blood draw and either symptom severity (spearman rho = 0.05, *p* = 0.75), or time to medical clearance (spearman rho = 0.17, *p* = 0.29), we observed a correlation between inflammatory biomarkers and days from SRC to blood draw (Supplementary Fig. 2). Lastly, our study focused on evaluating the relationship between symptom burden and systemic inflammation at a single time point post-injury, therefore follow-up studies are required to characterize this relationship throughout recovery; given our groups’ prior findings of persistent symptoms at medical clearance utilizing both advanced neuroimaging [63, 64] and blood biomarkers [65], it remains unclear how symptom burden tracks biological recovery.

## Conclusion

Symptom burden is associated with unique inflammatory profiles in male and female athletes following SRC. Symptom severity is associated with elevated blood concentrations of IFN-γ in males, yet lower levels of IFN-γ, TNF-α, and MPO in females: the relationship between symptoms and inflammation appears to be more generalizable in females. Future investigations into inflammation as a clinically meaningful secondary injury process following concussion should not ignore the potentially divergent pathophysiology underlying symptom presentation in males and females.

## Methods

### Participants

Participant eligibility and enrolment information are visualized in Fig. 1. From August 2015 – February 2018, 113 athletes with SRC were eligible for study enrollment. From this, 70 athletes were enrolled, and 49 consented to blood draw. Due to an inability to draw blood (*n* = 4), medications that interfered with inflammatory processes (*n* = 2), and an absence of SCAT symptom information (n = 3), 40 interuniversity athletes with a clinician diagnosed SRC were enrolled (*n* = 20 male (M), n = 20 female (F)) from nine sport teams: basketball (M & F), field hockey (F), football (M), ice hockey (M & F), lacrosse (M & F), mountain biking (F), rugby (M & F), soccer (F), volleyball (M & F). This cohort was analyzed in a previously published study by our group [6]. Concussion diagnosis and medical clearance decisions were made by a staff physician at the university sport medicine clinic in accordance with the Concussion in Sport Group guidelines [1]. Prior to enrollment, all participants provided written informed consent; all study procedures were in accordance with the declaration of Helsinki, and approved by the Health Science Research Ethics Board, University of Toronto (protocol reference # 27958).

### Blood biomarkers

Blood was sampled from athletes within a range of 2–7 days following an SRC (males, median = 4 days; females, median = 5 days). Athletes were excluded if they were taking medications other than birth control, or if they were currently symptomatic as a result of a known infection, illness or seasonal allergies. Venous blood was drawn into a 10-mL K_2_EDTA tube and was equilibrated for approximately one hour at room temperature before a two min centrifugation using a PlasmaPrep 12™ centrifuge (Separation Technology Inc., FL, USA). Plasma supernatant was then aliquoted and frozen at − 80 °C until analysis.

Nineteen cytokines and chemokines were analyzed by immunoassay using Meso Scale Diagnostics 96-well MULTI-SPOT® technology: interferon (IFN)-γ, interleukin (IL)-1β, − 2, − 4, − 6, − 8, − 10, −12p70, − 13, tumor necrosis factor (TNF)-α, eotaxin, eotaxin-3, interferon gamma-induced protein (IP)-10, monocyte chemoattractant protein (MCP)-1, − 4, macrophage-derived chemokine (MDC), macrophage inflammatory protein (MIP)-1α, −1β, and thymus and activation-regulated chemokine (TARC). Myeloperoxidase (MPO) was run as a single-plex assay. All assays were run according to manufacturer’s instructions, with individual samples run in duplicate.

### Symptoms

On the day of the blood draw, athletes’ concussion symptoms were ascertained via a 22-item post-concussion symptom scale using a seven-point Likert rating as part of the Sport Concussion Assessment Tool (SCAT). The SCAT is the most widely used tool to assist in the diagnosis, management, and prognosis of individuals with concussion [1], and has shown reliability and validity for the assessment of both symptom presence and severity [66, 67]. A total symptom score was obtained by summing the presence or absence of each symptom irrespective of severity, with a maximum value of 22; symptom severity was evaluated by summing the rated symptom score for each symptom. In addition, four distinct symptom clusters were obtained by combining and summing the scores of SCAT symptoms related to somatic complaints (headache, pressure in head, neck pain, nausea/vomiting, dizziness, blurred vision, balance problems, sensitivity to light, sensitivity to noise), cognition (feeling slowed down, feeling in a fog, don’t feel right, difficulty concentrating, difficulty remembering, confusion), sleep (fatigue/low energy, drowsiness, trouble falling asleep), and emotion (more emotional, irritability, sadness, nervous/anxious). This approach has been previously employed by our group [6, 68].

### Statistical analysis

Prior to statistical analysis, we applied a previously published set of biomarker detection criteria [6], retaining only biomarkers that contained individual values for > 80% of subjects per biomarker. Biomarker values were removed if they 1) did not fall within the manufacturer provided limits of detection, or 2) displayed a > 25% coefficient of variation between individual sample replicates. Hence, 10 of 20 inflammatory biomarkers satisfied these criteria and were evaluated in the current study. See Supplementary Table 1 for biomarker detection data in both male and female participant groups.

All variables (biomarkers and symptoms) were tested for deviations from normality by calculating sample skewness and kurtosis, with empirical *p*-values obtained by comparison against a simulated null distribution (random gaussian noise, 1000 simulated samples). In males, skewness ranged from 0 (*p* = 0.448) to 1.5 (*p* = 0.003), and kurtosis ranged from 3.2 (*p* = 0.721) to 9.4 (*p* = 0.001). In females, skewness ranged from – 0.9 (*p* = 0.949) to 2.2 (*p* < 0.001), and kurtosis ranged from 3.1 (*p* = 0.796) to 10.6 (p < 0.001). Hence, prior to all statistical comparisons, missing values were imputed separately in male and female subjects using the variable median, followed by rank transformation for symptom variables and a two-tailed winsorization (10%) of biomarkers that significantly deviated from normality.

Univariate comparisons of demographic variables and biomarkers between male and female athletes following SRC (Tables 1 & 2**)** were evaluated by calculating the mean differences, followed by bootstrapping of the mean difference scores (1000 resamples) to obtain standardized effect size in terms of bootstrap ratios (BSR; mean / standard error) and empirical *p* values based on the bootstrap estimates of the standard error, which were corrected at a false discovery rate (FDR) of 0.05. Univariate correlation analyses were conducted between calculated symptom severity and days to recovery via Spearman correlation with bootstrapping used to obtain BSRs and empirical p values.

The primary aim of the study was to test for associations between symptom reporting and inflammatory biomarker profiles in male and female athletes following SRC using a statistical framework designed to elucidate the potential complexity of these relationships, and without the potential confounds that would arise by including a comparison to a healthy athlete population. To accomplish this, a partial least squares (PLS) correlation analysis was employed [69]. PLS is a multivariate data reduction technique that creates orthogonal latent variables describing the maximal covariance between a set of predictor (biomarkers) and response (symptoms) variables [69]. In the current study, PLS was used in a bootstrap resampling framework (5000 iterations) to generate sets of variable loadings (i.e., weighted combinations of biomarkers / symptom clusters), along with corresponding BSRs and empirical *p*-values based on the bootstrap estimates of the standard error. For analyses evaluating the correlation between inflammatory biomarkers and calculated symptom severity, an out-of-sample, leave-two-out cross correlation (R^2^) value was calculated on the PLS model. PLS plots represent the mean and standard deviation (SD) of the resamples for each variable. All statistical analyses and graphs were completed with R (RStudio, version 1.1.456, Boston, United States).

## Supplementary information

**Additional file 1: Figure S1.** Correlation between inflammatory biomarkers and concussion history in male and female athletes following sport-related concussion. Eotaxin, interferon (IFN)-γ, interleukin (IL)-8, interferon gamma-induced protein (IP)-10, monocyte chemoattractant protein (MCP)-1, − 4, macrophage inflammatory protein (MIP)-1β, Myeloperoxidase (MPO), thymus and activation-regulated chemokine (TARC), and tumor necrosis factor (TNF)-α. Plots show the variable loadings in male (*n* = 20) and female (n = 20) athletes subacutely following a sport-related concussion, depicting their correlation to concussion history. Bars represent variable loadings and the standard error derived from bootstrapped resampling (5000 iterations, male = red, female = green). Significance is displayed at *p* < 0.05 *, *p* < 0.01** and *p* < 0.001***.

**Additional file 2: Figure S2.** Correlation between inflammatory biomarkers and days elapsed between sport-related concussion and blood draw. Eotaxin, interferon (IFN)-γ, interleukin (IL)-8, interferon gamma-induced protein (IP)-10, monocyte chemoattractant protein (MCP)-1, − 4, macrophage inflammatory protein (MIP)-1β, Myeloperoxidase (MPO), thymus and activation-regulated chemokine (TARC), and tumor necrosis factor (TNF)-α. Plots show the variable loadings in male (*n* = 20) and female (*n* = 20) athletes subacutely following a sport-related concussion, depicting their correlation to the number of days between injury and blood draw. Bars represent variable loadings and the standard error derived from bootstrapped resampling (5000 iterations, male = red, female = green). Significance is displayed at *p* < 0.05 *, *p* < 0.01** and *p* < 0.001***.

**Additional file 3: Table S1.** Biomarker Detectability.

## Data Availability

All data is available upon author request.

## Abbreviations

BBB: Blood brain barrier
BSR: Bootstrap ratio
CI: Confidence interval
CNS: Central nervous system
CRP: C-reactive protein
F: Female
HPA: Hypothalamic pituitary adrenal
IFN: Interferon
IL: Interleukin
IP: Interferon gamma induced protein
LV: Latent variable
M: Male
MCP: Monocyte chemoattractant protein
MDC: Macrophage-derived chemokine
MIP: Macrophage inflammatory protein
MPO: Myeloperoxidase
PCS: Post-concussion syndrome
PLS: Partial least squares
SCAT: Sport concussion assessment tool
SNS: Sympathetic nervous system
SRC: Sport-related concussion
TARC: Thymus and activation regulated chemokine
TBI: Traumatic brain injury
TNF: Tumor necrosis factor
