# Supplementary figures and images for: The relationship between symptom burden and systemic inflammation differs between male and female athletes following concussion
Source: BMC Immunol. 2020 Mar 12;21:11. doi: 10.1186/s12865-020-0339-3 (PMC7068899; doi:10.1186/s12865-020-0339-3)

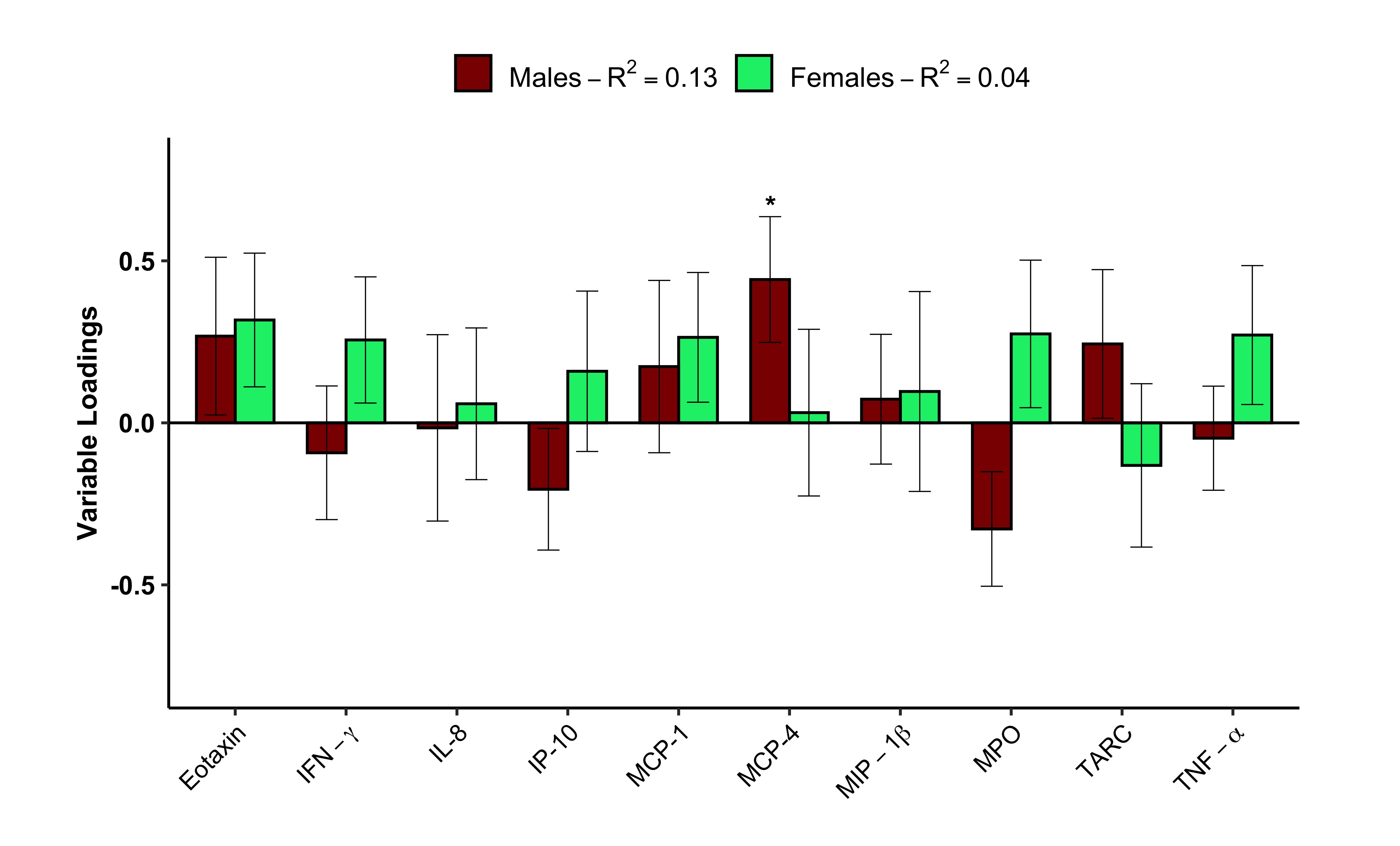

Supplement: Supplementary file 1 — Additional file 1: Figure S1. Correlation between inflammatory biomarkers and concussion history in male and female athletes following sport-related concussion. Eotaxin, interferon (IFN)-γ, interleukin (IL)-8, interferon gamma-induced protein (IP)-10, monocyte chemoattractant protein (MCP)-1, − 4, macrophage inflammatory protein (MIP)-1β, Myeloperoxidase (MPO), thymus and activation-regulated chemokine (TARC), and tumor necrosis factor (TNF)-α. Plots show the variable loadings in male (n = 20) and female (n = 20) athletes subacutely following a sport-related concussion, depicting their correlation to concussion history. Bars represent variable loadings and the standard error derived from bootstrapped resampling (5000 iterations, male = red, female = green). Significance is displayed at p < 0.05 *, p < 0.01** and p < 0.001***. [file 12865_2020_339_MOESM1_ESM.jpg]

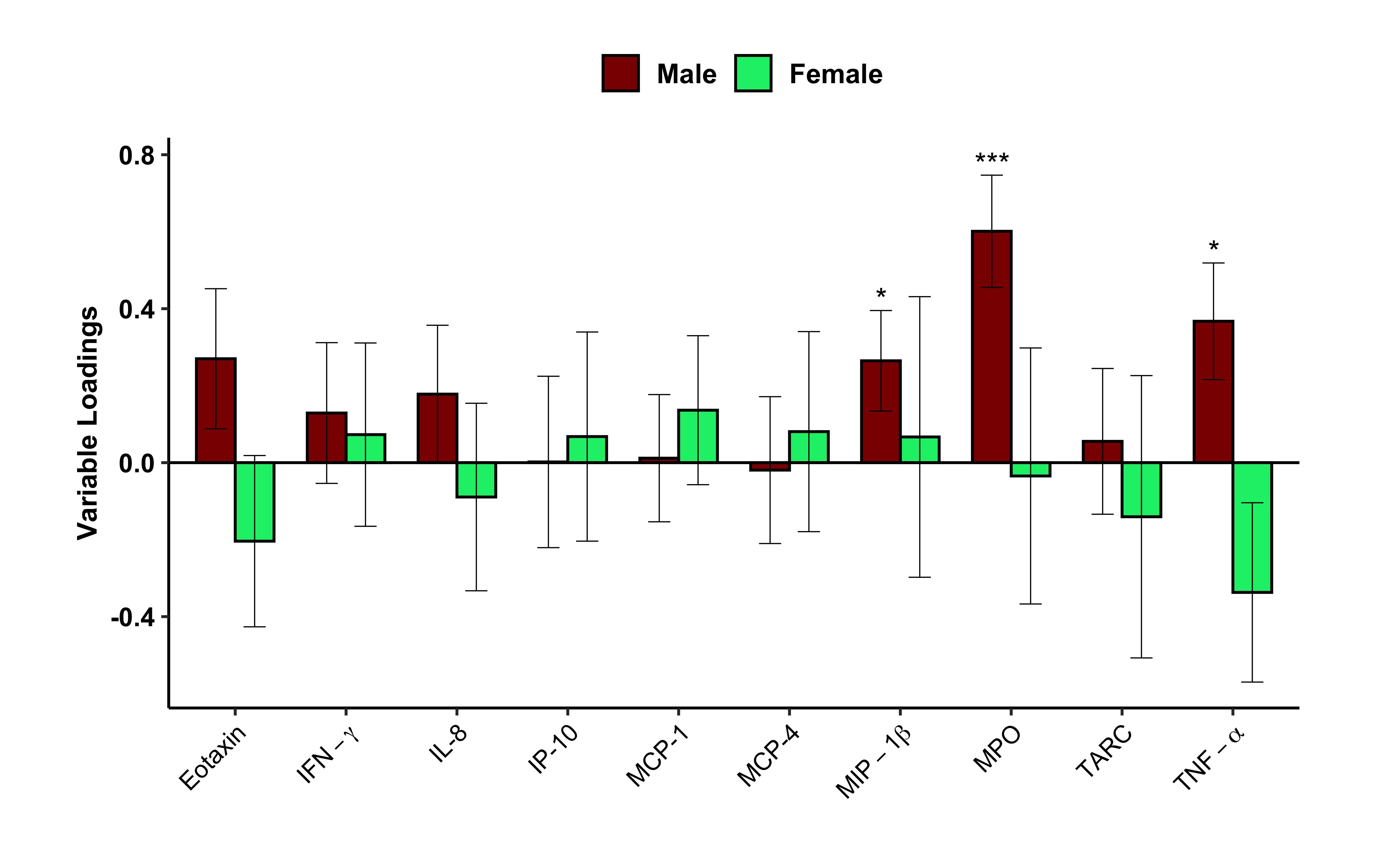

Supplement: Supplementary file 2 — Additional file 2: Figure S2. Correlation between inflammatory biomarkers and days elapsed between sport-related concussion and blood draw. Eotaxin, interferon (IFN)-γ, interleukin (IL)-8, interferon gamma-induced protein (IP)-10, monocyte chemoattractant protein (MCP)-1, − 4, macrophage inflammatory protein (MIP)-1β, Myeloperoxidase (MPO), thymus and activation-regulated chemokine (TARC), and tumor necrosis factor (TNF)-α. Plots show the variable loadings in male (n = 20) and female (n = 20) athletes subacutely following a sport-related concussion, depicting their correlation to the number of days between injury and blood draw. Bars represent variable loadings and the standard error derived from bootstrapped resampling (5000 iterations, male = red, female = green). Significance is displayed at p < 0.05 *, p < 0.01** and p < 0.001***. [file 12865_2020_339_MOESM2_ESM.jpg]
