# Supplementary material for: The relationship between symptom burden and systemic inflammation differs between male and female athletes following concussion
Source: BMC Immunol. 2020 Mar 12;21:11. doi: 10.1186/s12865-020-0339-3 (PMC7068899; doi:10.1186/s12865-020-0339-3)
Supplement: Supplementary file 3 — Additional file 3: Table S1. Biomarker Detectability. [file 12865_2020_339_MOESM3_ESM.docx]

**Supplementary Table 1.** Biomarker Detectability

| **Biomarker** | **Males (n = 20)** | **Females (n = 20)** |
| --- | --- | --- |
| IFN-γ | 20 (100) | 17 (85) |
| IL-1β | 3 (15) | 0 (0) |
| IL-2 | 4 (20) | 5 (25) |
| IL-4 | 1 (5) | 1 (5) |
| IL-6 | 7 (35) | 8 (40) |
| IL-8 | 20 (100) | 20 (100) |
| IL-10 | 13 (65) | 12 (60) |
| IL-12p70 | 4 (20) | 4 (20) |
| IL-13 | 0 (0) | 3 (15) |
| TNF-α | 20 (100) | 20 (100) |
| MPO | 19 (95) | 18 (90) |
| MCP-1 | 20 (100) | 20 (100) |
| MCP-4 | 20 (100) | 18 (90) |
| MIP-1α | 8 (40) | 10 (50) |
| MIP-1β | 20 (100) | 20 (100) |
| MDC | 8 (40) | 12 (60) |
| IP-10 | 20 (100) | 20 (100) |
| TARC | 18 (90) | 20 (100) |
| Eotaxin | 20 (100) | 18 (90) |
| Eotaxin-3 | 16 (80) | 13 (65) |

Values presented as the number and percent; n (%)

interferon (IFN)-γ, interleukin (IL), tumor necrosis factor (TNF)-α, myeloperoxidase (MPO), monocyte chemoattractant protein (MCP)-1, -4, macrophage inflammatory protein (MIP)-1α, -1β, macrophage derived chemokine (MDC), interferon gamma-induced protein (IP)-10, thymus and activation-regulated chemokine (TARC).
